# Rapid and Accurate Species-Specific PCR for the Identification of Lethal *Chironex* Box Jellyfish in Thailand

**DOI:** 10.3390/ijerph18010219

**Published:** 2020-12-30

**Authors:** Nuankanya Sathirapongsasuti, Kasetsin Khonchom, Thunyaporn Poonsawat, Mitila Pransilpa, Supaporn Ongsara, Usawadee Detsri, Suwimon Bungbai, Sam-ang Lawanangkoon, Worawut Pattanaporkrattana, Satariya Trakulsrichai

**Affiliations:** 1 Section of Translational Medicine, Faculty of Medicine Ramathibodi Hospital, Mahidol University, Bangkok 10400, Thailand; kasetsin.khn@mahidol.edu; 2 Marine and Coastal Resources Research Center, Central Gulf of Thailand, Chumphon 86000, Thailand; thunya-flower@hotmail.com; 3 Marine and Coastal Resources Research Center, Lower Gulf of Thailand, Songkhla 90100, Thailand; 4 Marine and Coastal Resources Research Center, Eastern Gulf of Thailand, Rayong 21170, Thailand; mitila.pransilpa@gmail.com; 5 Marine and Coastal Resources Research Center, Lower Andaman, Trang 92150, Thailand; cin_dmcr@hotmail.com (S.O.); dusawadee@gmail.com (U.D.); 6 Phuket Marine Biological Center, Phuket 83000, Thailand; 7 Koh Kut Hospital, Trat 23170, Thailand; suwimon0507@gmail.com; 8 Koh Phangan Hospital, Surat Thani 84280, Thailand; sam-ang_31@hotmail.com (S.-a.L.); baanhinnok@gmail.com (W.P.); 9 Ramathibodi Poison Center, Faculty of Medicine, Ramathibodi Hospital, Mahidol University, Bangkok 10400, Thailand; satariya.tra@mahidol.ac.th; 10 Department of Emergency Medicine, Faculty of Medicine, Ramathibodi Hospital, Mahidol University, Bangkok 10400, Thailand

**Keywords:** *Chironex*, box jellyfish, species identification, species-specific PCR

## Abstract

Box jellyfish are extremely potent venom-producing marine organisms. While they have been found worldwide, the highest health burden has been anticipated to be the tropical Indo-Pacific of Southeast Asia (SEA). At least 12 Cubozoan species have now been documented in Thai waters, and many of them inflict acutely lethal strings, especially those under the order Chirodropida. Our previous study has successfully differentiated species of box jellyfish using DNA sequencing to support the morphological study. In this study, we specifically designed polymerase chain reaction (PCR) primers for the 16S ribosomal RNA (rRNA) gene and the mitochondrial DNA cytochrome oxidase subunit I (COI) gene of lethal Thai *Chironex* species. The SYBR green-based real-time PCR panel was performed for rapid species identification. The sensitivity and specificity of the panel were determined by testing samples of different species. Moreover, we applied the panel to the tentacle sample from a real patient, which helped confirm the animal-of-cause of envenomation. Our results show a success for species identification of box jellyfish using 16S rRNA and COI PCR panel, which revealed congruence between molecular and morphological identification. Furthermore, the panel worked very well with the unknown samples and jellyfish tissue from the real envenomation case. The results demonstrated that molecular panels were able to identify three species of *Chironex* box jellyfish both rapidly and accurately, and can be performed without having a complete specimen or morphological study.

## 1. Introduction

Class Cubozoa, phylum Cnidaria, often referred to as box jellyfish [1,2], are extremely potent venom-producing marine organisms. Many box jellyfish species inflict acutely lethal stings (order Chirodropida) and represent significant marine morbidity and mortality threat in tropical and subtropical waters. However, studying the box jellyfish is challenging according to their logistical difficulties: Habitat in remote locations, patchy distribution, low abundance, dangerous, and challenging sampling [3]. Moreover, in many cases, only parts of the animal, mostly tentacles, were found. Since marine biodiversity study still uses morphological analysis as a principal species identification tool that requires a complete specimen and should be done by well-trained taxonomists. Furthermore, there is increasing evidence of cryptic species within jellyfish’s clades that are indistinguishable from morphology [4,5,6], making traditional species identification even more difficult.

To date, over 50 species have been identified [2]. While distributing globally, we believe that box jellyfish have focal habitats in Southeast Asian (SEA) waters according to progressively discovered species and sting cases reports [7,8,9,10,11,12]. In Thailand, for instance, based on the Thai Department of Marine and Coastal Resources (DMCR) and the international efforts, at least 12 different species of box jellyfish have been documented in Thai waters [13,14]: Three *Chironex* species, *Chiropsoides buitendijki* (van der Horst, 1907), *Meteorona kishinouyei* Toshino, Miyake and Shibata, 2015., three *Morbakka* species, *Copula sivickisi* (Stiasny, 1926), *Tripedalia cystophora* Conant, 1897, *Tripedalia binata* Moore, 1988, and *Alatina morandinii* (Straehler–Pohl and Jarms, 2011). Of these, three *Chironex* species are suspected to be the potential cause of life-threatening or severe envenomation in Thailand [10,11,12]. Regarding to what we have learned from Thailand, highly diverse Cubozoan species are expected in SEA. SEA is one of the top tourist destinations, especially for coastal and marine activities. Thus, a rapid species-identification will help not only for proper clinical management but also for proficient marine safety and injury prevention.

Molecular identification techniques: Polymerase chain reaction (PCR), real-time PCR, and DNA sequencing are more available and affordable, which have become mainstay methods in biology, especially in cryptic species identification. In this study, based on our sequencing data from previous work [15] on DNA barcoding of 16S rRNA, 18S rRNA, and cytochrome oxidase subunit I (COI) genes of nine Thai box jellyfish species, we individually designed species-specific primers for rapid and accurate PCR-based molecular identification.

## 2. Materials and Methods

### 2.1. Box Jellyfish Specimens

Fifty samples from nine species of box jellyfish tentacles, listed in Table 1, were collected from Thai waters according to the approved protocol, Mahidol University-Institute Animal Care and Use Committee, and Mahidol University Biosafety Committee. Fresh tentacles were cut and kept in absolute ethanol at 4-degree Celsius. The rest of the animals were kept in 3% formaldehyde for morphological identification.

For panel validation, seventeen tentacle sample s from unknown Chirodropid species, no morphological study, were accumulated by Koh Phangan Hospital, five tentacle samples of Chirodropids from Phuket Marine Biological Center, four tentacle samples of Chirodropids from the Marine and Coastal Resources Research Center, Central Gulf of Thailand, twelve tentacle samples of Chirodropids from the Marine and Coastal Resources Research Center, Eastern Gulf of Thailand, and one tentacle sample collected from near-fatal envenomation patient’s skin from Koh Kut Hospital, were collected for species identification.

### 2.2. DNA Extraction, PCR, and Sequencing

Genomic DNA was extracted from tentacle tissue by using the QIAamp DNA Mini Kit (Qiagen, Germany) according to the manufacturer’s instructions. A pair of universal 16S rRNA primers P16sf (5’AAGGGCCGCGGTAACTCTG 3’) and S16sr (5’ ACCCTGTTATCCCCGTGGT 3’) [16] were used to amplify the 16S rRNA gene. The universal COI primers jgHCO2198 (5’-TANACYTCNGGRTGNCCRAARAAYCA-3’) and jgLCO1490 (5’-TNTCNACNAAYCAYAARGAYATTGG-3’) [17] were used for COI amplification. PCRs were performed in a final volume of 50 ul containing 50 ng of DNA as a template, 10 uM each primer, a 25 mM concentration of each deoxynucleoside triphosphate, and 5 U of Taq polymerase. The PCR protocol for 16S rRNA gene included an initial denaturing step at 95 °C for 5 min, followed by 30 cycles of 95 °C for 30 s, 64 °C for 30 s and 72 °C for 1 min with a final extension at 72 °C for 5 min. While, the PCR protocol for COI gene included an initial denaturing step at 94 °C for 2 min, followed by 30 cycles of 92 °C for 40 s, 45 °C for 40 s and 72 °C for 1 min 30 s with a final extension at 72 °C for 5 min. The reactions were performed on a MyCycler Thermal Cycler (Bio-Rad Laboratories, Inc., Hercules, CA, USA). The PCR products were then visualized by gel electrophoresis on a 1.5% (*w*/*v*) agarose gel in 1X Tris acetate-ethylene diamine tetra-acetic acid (EDTA) buffer, stained with ethidium bromide and visualized with a UV light (The Gel Doc XR+ System, Bio-Rad Laboratories, Inc.). DNA purification was performed using PCR Purification Kit (Qiagen, Germany). The bidirectional sequencing using the same amplification primers was performed by Macrogen (Seoul, Korea) and Bioneer (Daejeon, Korea).

### 2.3. Sequence Analysis

The three *Chironex* species 16S rRNA and COI gene contigs were assembled from forward and reverse reads of each 16S rRNA and COI amplicon. MAFFT software (version 7) [18,19] was used for multiple DNA alignment. Sixty-eight amplicon sequences, 62 from laboratory and 6 from Genbank, were aligned. The phylogenetic trees were constructed using Molecular Evolutionary Genetics Analysis version 7.0 (MEGA7) [20].

### 2.4. Species-Specific Primers Design, Selection, and Validation

Suitable areas for designing species-specific primers were identified with Multiple alignment program for amino acid or nucleotide sequences (MAFFT) version 7 [18,19], and species-specific primers for *Chironex* identification were designed with Aliview software (version 1.26) [21], OligoAnalyzer software (Integrated Device Technology, Inc., San Jose, CA, USA), and Primer-BLAST software (NCBI National Center for Biotechnology Information, U.S.). Primer pairs were evaluated according to 7 factors: (1) length between 18 bp to 33 bp; (2) 3′-end contains one or more specific bases; (3) Delta G for hairpin less than 1 kcal/mole; (4) GC% from 35% to 60%; (5) primers for distinguishing different species; (6) false priming less than 100%; and (7) optimal annealing temperatures. All of the primers were synthesized by Integrated Device Technology, Inc.

Specificity testing with each primer pair in the SYBR green-based real-time PCR assays was performed using 50 samples of 9 species of box jellyfish found in Thai waters (Table 1). PCR amplification in a final reaction volume of 10 μL consisted of 5 μL SYBR Green master mix, 3 μL ddH2O, 0.5 μL specific forward primer, 0.5 μL specific reverse primer, and 1 μL template DNA. The PCR cycler conditions for 16S rRNA gene used were an initial denaturation at 95 °C for 120 s, followed by 30 cycles of 95 °C for 15 s, and 64 °C for 60 s. The PCR cycler conditions for COI gene were an initial denaturation at 95 °C for 120 s, followed by 30 cycles of 95 °C for 15 s, 55 °C for 30 s, and a final extension at 72 °C for 1 min. The PCR products were then visualized by gel electrophoresis on a 1.5% (*w*/*v*) agarose gel in 1X Tris acetate-EDTA buffer, stained with ethidium bromide and visualized with a UV light (The Gel Doc XR+ System, Bio-Rad Laboratories, Inc.).

Sensitivity testing was determined in PCR runs with a series of decreasing sample DNA concentrations of 100, 10, 1, 0.1, 0.01, and 0.001 ng μL^−1^. Two replicates of each treatment were tested.

## 3. Results

### 3.1. Three Chironex Box Jellyfish Found in Various Locations in Thailand

**Figure 1:**
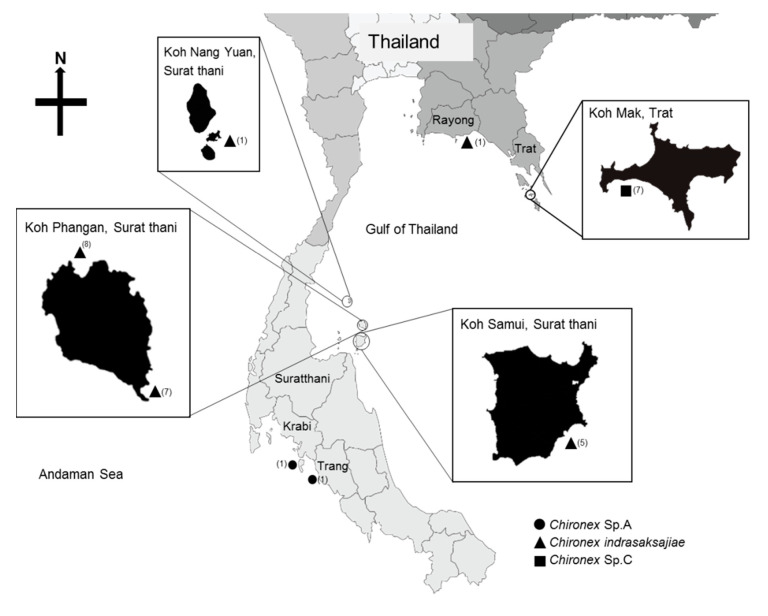
Map of Thailand, showing the species and locations where *Chironex* samples used in this study were found. The numbers in the bracket are animal numbers collected from each location.

Thirty-one complete samples of three *Chironex* species; *Chironex indrasaksajiae* (n = 22); *Chironex* Sp.A (n = 2); *Chironex* Sp.C (n = 7), were collected from various locations, as shown in Figure 1. The species of each sample was determined based on morphological anatomy [13].

### 3.2. Amplification and Sequencing Analysis of the 16S rRNA and COI Genes

**Figure 2:**
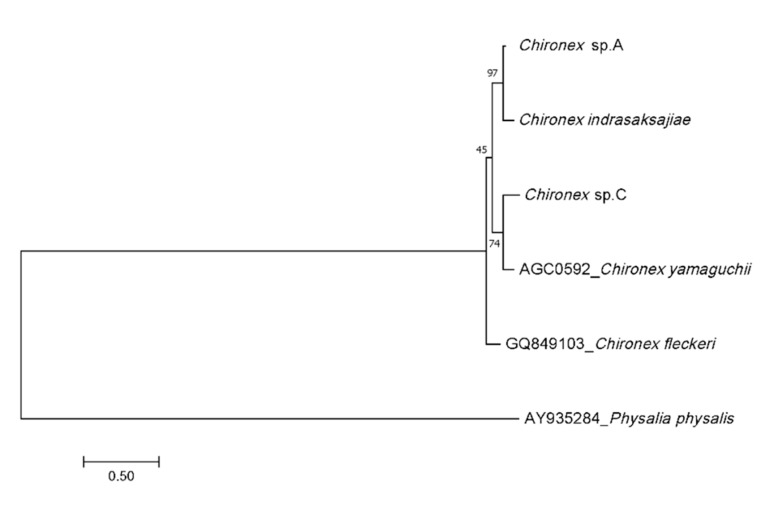
Phylogenetic tree of *Chironex* species based on the 16S rRNA gene sequences.

**Figure 3:**
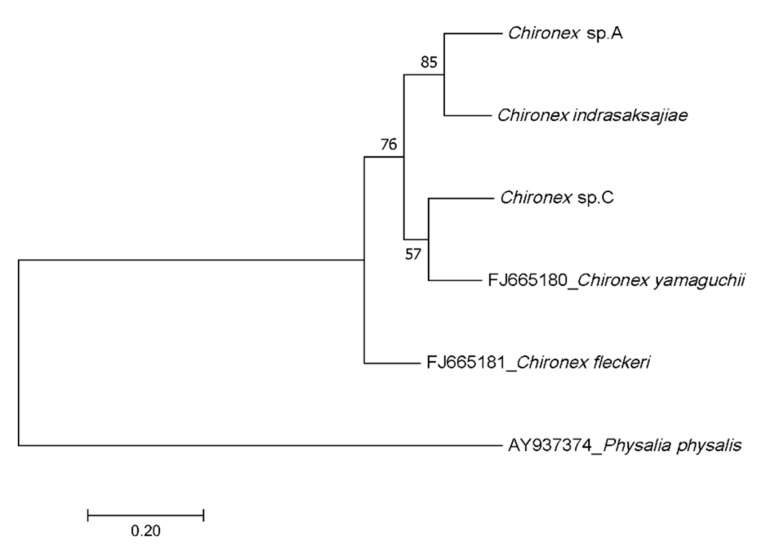
Phylogenetic tree of *Chironex* species based on the COI gene sequences.

The 414 bp and 709 bp long 16S rRNA and mtDNA COI genes were successfully amplified from 31 *Chironex* samples using Cubozoa universal primers. Direct sequencing of the amplicons was performed by Sanger sequencing. Unrooted phylogenetic trees, based on 62 sequences, 6 *Chironex* sequences from GeneBank, and one outgroup species *Physalia physalis* (Table 2), showed three distinct, well-supported morphological identification (Figure 2 and Figure 3). The phylogenetic trees obtained for 16S and COI segments showed that *Chironex* Sp.A clustered with *Chironex indrasaksajiae* and *Chironex* Sp.C clustered with *Chironex yamaguchii*, which congruence with the morphology. However, *Chironex indrasaksajiae* and *Chironex* Sp.A share sequence similarity of 92.03% in 16S rRNA and 86.33% in COI, while *Chironex yamaguchii* and *Chironex* Sp.C share sequence similarity of 88.14% in 16S rRNA and 85.43% in COI gene.

### 3.3. Species-Specific Primer Design for Three Thai Chironex Species

**Figure 4:**
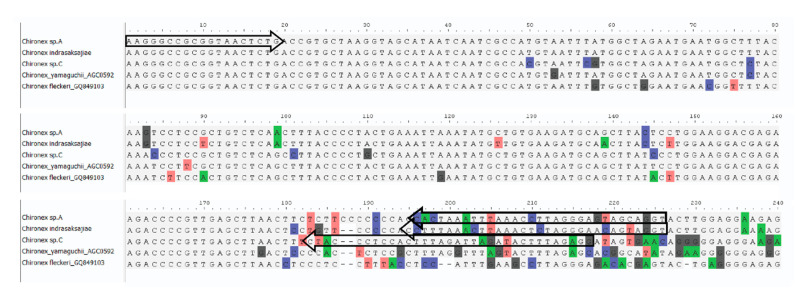
16S rRNA sequence alignment from five *Chironex* species. The forward arrows are forward primers; the reverse arrows are reverse primers.

**Figure 5:**
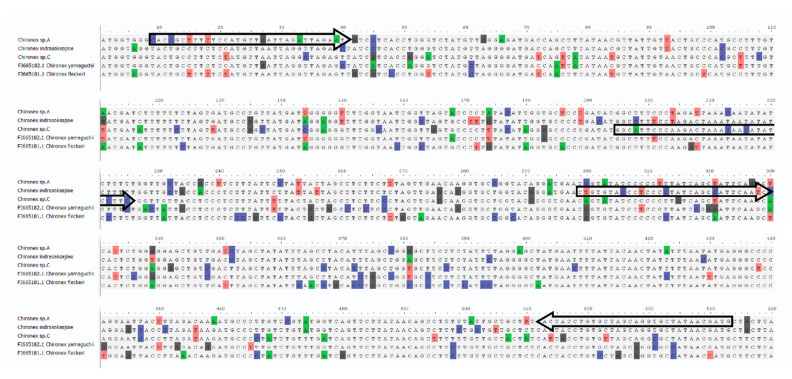
COI sequence alignment from five *Chironex* species. The forward arrows are forward primers; the reverse arrows are reverse primers.

Multiple alignments of 34 16S rRNA and 34 COI sequences were performed by MAFFT version 7 program to identify suitable regions for primer design (Figure 4 and Figure 5). The universal forward primer was taken from Sucharitakul et al. [16] and three species-specific reverse primers were newly designed for 16S rRNA (Table 3). On the other hand, species-specific forward primers and universal reverse primer were designed for COI (Table 4).

**Figure 6:**
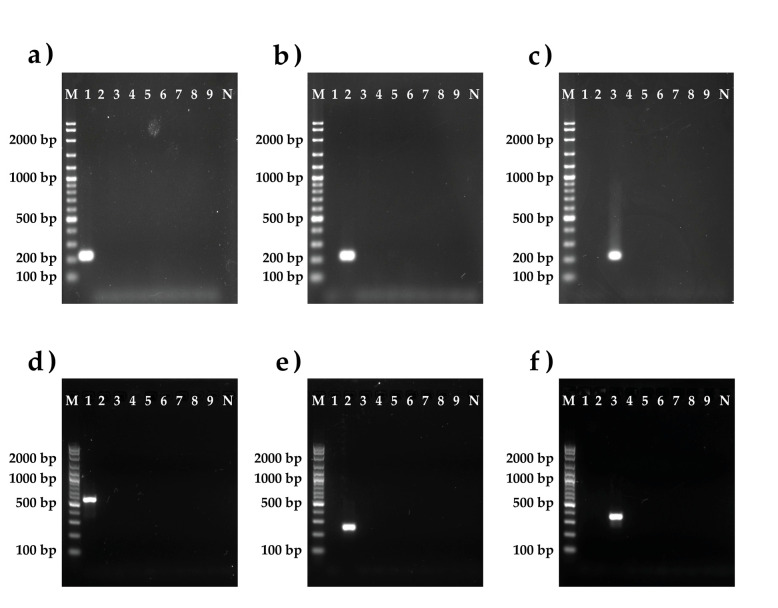
Gels from six PCR reactions validating the species specificity of the designed primers for three *Chironex* species. (**a**) 16S rRNA gene specific primers for *Chironex* Sp.A. (**b**) 16S rRNA gene Specific primers for *Chironex indrasaksajiae*. (**c**) 16S rRNA gene specific primers for *Chironex* Sp.C. (**d**) COI gene specific primers for *Chironex* Sp.A. (**e**) COI gene specific primers for *Chironex indrasaksajiae*. (**f**) COI gene specific primers for *Chironex* Sp.C. M: VC 100 bp Plus DNA Ladder (Vivantis, Malaysia); 1 *Chironex* Sp.A; 2 *Chironex indrasaksajiae*; 3 *Chironex* Sp.C; 4 *Morbakka* Sp.A; 5 *Morbakka* Sp.B; 6 *Morbakka* Sp.C; 7 *Meteorona* spp.; 8 *Chiropsoide buitendijki*; 9 *Copula sivickisi*.; and N no template.

The specificity tests were performed by uniplex SYBR green-based real-time PCR. The primer specificity was confirmed by the presence of a clear band on gel electrophoresis (Figure 6). For possible cross-reactivity with other Cubozoan species, we additionally checked the designed primers with 19 samples from six species of Thai box jellyfish: *Chiropsoides buitendijki*, *Meterona* spp., *Morbakka* Sp.A, *Morbakka* Sp.B, *Morbakka* Sp.C, and *Copula sivickisi*. The results clearly indicate that each pair of primers identified *Chironex* species specifically and showed no cross-reactivity with other Thai Cubozoan species.

**Figure 7:**
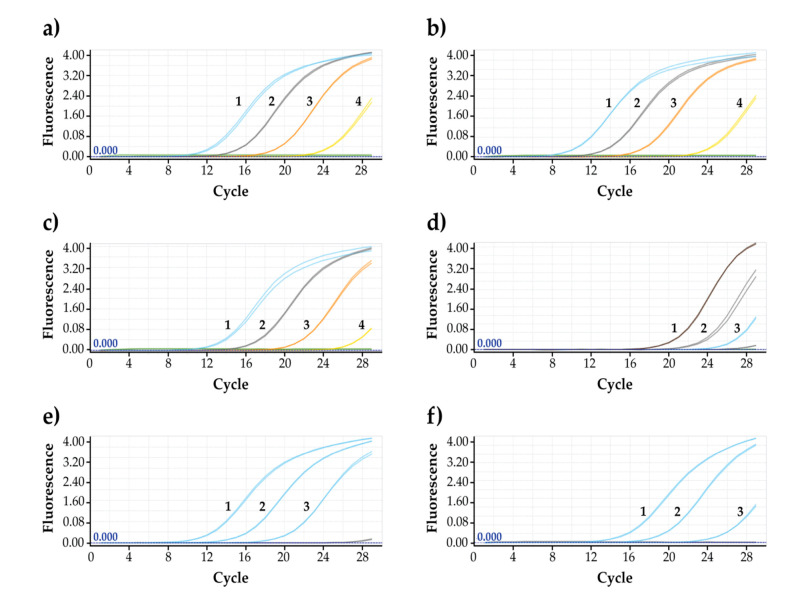
Sensitivity of the designed primers for three *Chironex* species (amplification curves). (**a**) 16S rRNA gene for *Chironex* Sp.A. (**b**) 16S rRNA gene for *Chironex indrasaksajiae*. (**c**) 16S rRNA gene for *Chironex* Sp.C. (**d**) COI gene for *Chironex* Sp.A. (**e**) COI gene for *Chironex indrasaksajiae*. (**f**) COI gene for *Chironex* Sp.C. 1: 10 ng; 2: 1 ng; 3: 0.1 ng; 4: and 0.01 ng (DNA template concentrations).

Sensitivity of the PCR panels was determined by varying the starting DNA materials of 10, 1, 0.1, and 0.01 ng. For all three *Chironex* species, the lower limit of detection was 0.01 ng for COI gene (Figure 7). On the other hand, for 16S rRNA, even a low amount of DNA template resulted in strong positive signals (Figure 7).

### 3.4. Panel Validation with Unknown Species and Real Clinical Samples

Seventeen tentacle samples from unknown Chirodropid species with no morphological study were accumulated by Koh Phangan Hospital; blind samples of five tentacle samples of Chirodropids from Phuket Marine Biological Center, four tentacle samples of Chirodropids from the Marine and Coastal Resources Research Center, Central Gulf of Thailand, and twelve tentacle samples of Chirodropids from the Marine and Coastal Resources Research Center, Eastern Gulf of Thailand; and one tentacle sample collected from near-fatal envenomation patient’s skin from Koh Kut Hospital were used for PCR panel validation. The species of all samples were successfully identified: All seventeen samples from Koh Phangan hospital were positive for *Chironex indrasaksajiae*; five samples from Phuket Marine Biological Center were positive for *Chironex* Sp.A, three samples from the Marine and Coastal Resources Research Center, Central Gulf of Thailand were positive for *Chironex indrasaksajiae,* and one sample was positive for *Chironex* Sp.C; all twelve samples from the Marine and Coastal Resources Research Center, Eastern Gulf of Thailand were positive for *Chironex* Sp.C; and the jellyfish sample on patient skin was positive for *Chironex indrasaksajiae.*

## 4. Discussion

Cubozoan or box jellyfish is the most venomous creature on the planet. While globally, they are believed to be most prevalent in the tropical Indo-Pacific region, especially in SEA. Thus, public health and tourist safety dictate that concerted actions to effectively address this issue are now many SEA national priorities. In Thailand, at least twelve species of box jellyfish have been identified, in which *Chironex* species was found to have potent venom and, therefore, the capacity to kill. To date, *Chironex fleckeri*, the multi-tentacle box jellyfish found in Australia, has been described as “the most lethal jellyfish in the world.”. But from reviewed clinical data in Thailand, we found that *Chironex indrasaksajiae*, newly identified box jellyfish from Thai waters have more potent venom than *Chironex fleckeri* by causing the systemic signs and symptoms within 1 min and cardiac arrest or death within 2–3 min [10,16]. According to what we have learned from Thailand, highly diverse Cubozoan species are expected in SEA. Moreover, since there is no territory in the water, animals can move around freely, especially box jellyfish that have the ability to swim up to 4 knots [22]. There is a high possibility that the animals found in Thai waters are also found in neighboring countries. For example, the *Chironex* species that we found in our Andaman Sea might also be found and could be a cause for fatal cases in Langkawi; *Chironex* species found in the Eastern Gulf of Thailand might be found in Cambodian or Vietnamese waters as well. Despite increasing envenomation cases and health burden in the regions, expertise related to box jellyfish: Animal survey, species identification, distribution, predictable seasonal, and geographic hot spots, is very limited.

This study was the first and perhaps the most extensive Cubozoan DNA collection in SEA. The partial sequences of 16S rRNA and COI were analyzed to identify suitable regions for species-specific primers. We successfully attained a PCR panel that could identify *Chironex* species specifically with no cross-reactivity with other Thai Cubozoan species. Since PCR and real-time PCR have become a standard technique in the laboratory. We believe that this rapid and accurate PCR-based *Chironex* species identification panel would help identify the cause of envenomation for proper clinical management and facilitate the box jellyfish research and enhance better awareness and insight that would lead to proficient marine safety and injury prevention in the SEA regions. Moreover, we found two out of three Thai *Chironex* box jellyfish, *Chironex indrasaksajiae* and *Chironex* Sp.A, are morphologically similar but genetically different. They would be the sister or cryptic species. To further scrutinize this, deep sequencing to compare the whole genome sequences would help us trace back these animals’ origin and evolution. Without the complete genome sequencing and comparative genomic analysis, this study showed a relatively simple way to identify sister or cryptic species using two molecular markers: 16S rRNA and the mitochondrial DNA COI gene. However, because the panel was designed explicitly for Thai *Chironex* species, it may or may not be valid for *Chironex* species elsewhere. We believe that our approach could be used as an exemplar for other unknown or new species.

## 5. Conclusions

Molecular biology assays have been well implemented especially for molecular identification. In this study, a series of experiments have been done with the objective to develop a PCR-based strategy for rapid and accurate species identification of *Chironex* box jellyfish found in Thai waters. Our results show a success for species identification of box jellyfish using 16S rRNA and COI PCR panel, both conventional PCR and SYBR green-based real-time PCR assays. Furthermore, the panel worked very well with the unknown samples and jellyfish tissue from the real envenomation case. The results demonstrated that molecular panels were able to identify three species of *Chironex* box jellyfish both rapidly and accurately. In addition, it could be performed without having a complete sample or morphological study.

## Figures and Tables

**Table 1:** The box jellyfish specimens used in this study. Fifty samples from nine species of Thai box jellyfish were collected for morphological and molecular analysis.

Order	Family	Species	Number of Animals
Chirodropida	Chirodropidae	*Chironex indrasaksajiae*	22
		*Chironex* Sp.A	2
		*Chironex* Sp.C	7
	Chiropsalmidae	*Chiropsoides buitendijki*	2
	Chiropsellidae	*Meteorona kishinouyei*	6
Carybdeida	Carukiidae	*Morbakka* Sp.A	3
		*Morbakka* Sp.B	4
		*Morbakka* Sp.C	2
	Tripedaliidae	*Copula sivickisi*	2
Total			50

**Table 2:** The information of 16S rRNA and cytochrome oxidase subunit I (COI) gene sequences from GenBank.

Species	Accession No.	
	16S	COI
*Chironex fleckeri*	GQ849103	FJ665181
*Chironex yamaguchii*	AGC0592	FJ665180
*Chironex indrasaksajiae*	KX090147	KT223648
*Physalia physalis*	AY935284	AY937374

**Table 3:** List of the 16S rRNA primers of three Thai *Chironex* species.

Sample	Name	Seq. (5’ → 3’)	Length (bp)	Tm (°C)	Product (bp)
Universal primer	P16sf	AAGGGCCGCGGTAACTCTG	19	61.7	414
	S16sr	ACCCTGTTATCCCCGTGGT	19	59.5	
*Chironex* Sp.A	P16sf	AAGGGCCGCGGTAACTCTG	19	61.7	226
	CA16sr	ACCTGCTACTCCCTAAGGTTTAAATTTAGTG	31	58	
*Chironex indrasaksajiae*	P16sf	AAGGGCCGCGGTAACTCTG	19	61.7	226
	CI16sr	ACCTACTGTTCCCTAGAGTTTAAGTTTAAAGG	32	57.6	
*Chironex* Sp.C	P16sf	AAGGGCCGCGGTAACTCTG	19	61.7	217
	CC16sr	CCTCTAAAGTATCTAATCTAAAGTGGAGGGTAG	33	57	

**Table 4:** List of the COI primers of three Thai *Chironex* species.

Sample	Name	Seq. (5’ → 3’)	Length (bp)	Tm (°C)	Product (bp)
Universal primer	jgLCO1490	TNTCNACNAAYCAYAARGAYATTGG	25	56	709
	jgHCO2198	TANACYTCNGGRTGNCCRAARAAYCA	26	60	
*Chironex* Sp. A	CAcof	CACCGCTTTTTCCATGTTGATTAGATTAGAAT	32	57.7	535
	Chironex_cor	CATCGTTATAGCRCCTGCTARCACAGGYART	31	62.8	
*Chironex indrasaksajiae*	CIcof	CTGTGTACCCTCCCCTATCAGCCATTCAATC	31	62.8	245
	Chironex_cor	CATCGTTATAGCRCCTGCTARCACAGGYART	31	62.8	
*Chironex* Sp.C	CCcof	GGCATTCCCAAGACTAAACAACATATCCTTC	31	59.1	349
	Chironex_cor	CATCGTTATAGCRCCTGCTARCACAGGYART	31	62.8	

## Data Availability

The data presented and generated during the study are publicly available. The data can be found here: [https://med.mahidol.ac.th/transmed/en/Research/NS/Research/]
